# Impact of COVID-19 on heart rate variability in post-COVID individuals compared to a control group

**DOI:** 10.1038/s41598-024-82411-w

**Published:** 2024-12-28

**Authors:** Aldair Darlan Santos-de-Araújo, Daniela Bassi-Dibai, Renan Shida Marinho, Izadora Moraes Dourado, Lucivalda Viegas de Almeida, Sigrid de Sousa dos Santos, Shane A. Phillips, Audrey Borghi-Silva

**Affiliations:** 1 Cardiopulmonary Physiotherapy Laboratory, Physical Therapy Department, Universidade Federal de São Carlos, Rodovia Washington Luiz, São Carlos, SP 13565-905 Brazil; 2 Department of Dentistry, Universidade CEUMA, São Luís, MA Brazil; 3 Postgraduate Program in Management in Health Programs and Services, Universidade CEUMA, São Luís, MA Brazil; 4 Postgraduate Program Inter-Units of Bioengineering, University of São Paulo, São Carlos, SP Brazil; 5 Medicine Departarment, Universidade Federal de São Carlos, São Carlos, SP Brazil; 6 Department of Physical Therapy, College of Applied Health Sciences, University of Illinois Chicago, Chicago, USA

**Keywords:** Autonomic nervous system, Heart rate variability, COVID-19, Computational biology and bioinformatics, Cardiovascular biology, Neurological disorders, Respiratory tract diseases, Viral infection

## Abstract

This study investigated the impact of mild COVID-19 on HRV in groups stratified by time after infection and to compare to a healthy group of the same age without previous virus infection and without need of hospitalization. This is a cross-sectional study. We divided the sample into four groups: control group (CG) (*n* = 31), group 1 (G1): ≤6 weeks (*n* = 34), group 2 (G2): 2–6 months (*n* = 30), group 3 (G3): 7–12 months (*n* = 35) after infection. For HRV analysis, we used the indices of linear (time and frequency domain) and non-linear analysis. For comparisons between groups, ANOVA one way test or Kruskal–Wallis was used according to the data distribution. The effect size was calculated based on Cohen’s d or η^2^. Simple and multiple linear regressions were performed to investigate the interaction between clinical outcomes and HRV parameters. A total of 130 individuals were included. Groups G1 and G2 showed less parasympathetic modulation when compared to CG (*p* < 0.05), while G3 showed an increase in parasympathetic modulation when compared to G1 (*p* < 0.05). Moderate to large effect sizes were found according to Cohen d or η^2^. The multiple linear regression models identified age and infection duration as significant predictors for RMSSD (adjusted R^2^ = 0.227) and SD1 (adjusted R^2^ = 0.242), while age was significant for SDNN (adjusted R^2^ = 0.213). BMI, hypertension, and dyslipidemia were non-significant in all models. For HF (n.u.), infection duration was consistently significant, with stress emerging as a predictor in Model 2 (adjusted R^2^ = 0.143). The recovery time since diagnosis and age influences recovery from HRV, suggesting a transient effect of the disease on the autonomic nervous system.

## Introduction

Heart rate variability (HRV) has endorsed a series of scientific publications that address the impairment of the autonomic nervous system (ANS) in individuals affected by COVID-19 at different levels of severity^1–3^. Beforehand, the results are inconclusive and elementary^4^, requiring further clarification regarding the impairment of this system, however, the storm of inflammatory cytokines triggered by this disease contributes not only to the impairment of homeostasis but also partially justifies the reason for the disturbance from ANS^2,5,6^. This, once affected, triggers an imbalance in the ANS, requiring an organic compensation response to deal with the systemic impairment in an attempt to preserve homeostasis^2^.

The relationship between viral infection and HRV has been investigated in various pathological conditions and ANS impairment has been documented at different levels of impairment and severity^7–9^. However, regarding to COVID infection, what must be reported today is that science has experienced scientific divergences that contrast or an increase in parasympathetic versus sympathetic tone in HRV in individuals who have not been hospitalized, symptomatic or asymptomatic, in different age groups^1,10^. This ends up making it possible to open up a wide margin of discussion about the true nature of cardiac autonomic modulation in this population due to the scarcity of evidence, the wide variety of recording tools used, the recording time for subsequent analysis and the inconclusive data published at the time, leading to difficulty in understanding the real physiological mechanism to making decision in clinical practice^11^.

In view of this and aware of the scientific gap, this study aims to investigate the impact of COVID-19 on HRV in groups stratified by time after infection (≤ 6 weeks, 2–6 months and 7–12 months) when compared to a matched healthy controls. Secondarily, we aimed to investigate whether the time of infection influences on HRV variables. Our hypothesis is that individuals who had COVID-19 have a decrease in HRV with sympathetic predominance in the early stages of infection and that there is an association between HRV outcomes and the time since diagnosis of the infection.

## Methodology

### Study design and ethical considerations

A cross-sectional study reported in accordance with the guidelines of the Strengthening Reporting of Observational Studies in Epidemiology^12^. The research was conducted at the Universidade Ceuma (São Luís—MA, Brazil/report number: 4.179.747) and Universidade Federal de São Carlos (São Carlos—SP, Brazil/report number: 5.499.064) after the study procedures were approved by the Research Ethics Committee of the institution and conducted according to Declaration of Helsinki. All individuals were informed of the purpose of the study and informed consent was obtained.

### Participants

Individuals of both sexes, aged ≥ 18 years, positive for SARS-CoV-2 according to the positive reverse transcription–polymerase chain reaction (RT-PCR) test were included between November 2020 and September 2023. Mild symptoms of COVID-19 were defined in accordance with the National Institutes of Health’s COVID-19 Treatment Guidelines^13^: presence of signs and symptoms of the disease, such as fever, cough, sore throat, malaise, pain headache, muscle pain, nausea, vomiting, diarrhea, loss of taste and smell, peripheral oxygen saturation (≥ 95%), i.e., without the need for additional oxygen therapy and/or mechanical ventilation, shortness of breath (> 20 breaths for minute), who were non-hospitalized or presented abnormal chest image.

Individuals were stratified according to time of infection into three groups: Group 1 (G1): individuals who were evaluated within 6 weeks after testing positive for SARS-CoV-2 and completing the isolation period; Group 2 (G2) individuals who were evaluated between 2 and 6 months after infection; Group 3 (G3) individuals who were evaluated between 7 and 12 months after infection. Individuals were excluded if they were diagnosed with moderate, severe or critical illness, had an episode of myocardial infarction, implanted a pacemaker or any metal synthesis, had a history of heart disease, unstable angina, uncontrolled hypertension, uncontrolled diabetes mellitus, dependent insulin, chronic obstructive pulmonary disease, neoplasms, cognitive impairment, declared users of illicit drugs and pregnancy.

Control group: the inclusion criteria for this group were based on self-report. The participants in this group were recruited before the pandemic and are part of a pre-existing laboratory database. To meet the criteria, it was essential that apparently healthy individuals had no history of cardiovascular, respiratory, metabolic and/or systemic diseases (such as hypertension, diabetes mellitus, cardiac arrhythmias, hormonal disorders, respiratory diseases of a restrictive or obstructive nature, cancer, ongoing viral infection etc.), inflammation or pain of any nature, they could not present neurological disorders and cognitive deficits that threatened to impair the understanding and execution of assessments, they could not be smokers or ex-smokers, nor make continuous and disordered use of alcohol and/or drugs illicit. Additionally, individuals could not have taken psychotropic medications or other medications known to alter autonomic activity for at least 4 weeks prior to the start of the study.

### Sample size

We used post-hoc sampling from the G*Power family of F tests (version 3.1.9.7.) for one-way ANOVA^14^. The total sample should vary between 128 and 132 individuals divided into four independent groups with similar average ages (*p* > 0.05) and standard deviation between 10 and 13 points within each group^15,16^. As such, the strategy is composed of effect size F = 0.21, alpha = 0.05, lambda = 6.27, critical F = 2.67, and power (1–β) = 0.52^15,16^. Therefore, the study should have a sample size configuration of at least 130 participants divided into respective groups.

### Assessment of dyspnea—modified Medical Research Council (mMRC)

This instrument comprises a questionnaire with five items, wherein patients assess the extent of their disability, indicating the impact of dyspnea on their mobility. Patients express their subjective dyspnea level by selecting a value from 0 to 4^17^. Higher mMRC scores correlate with severe limitations in daily activities associated with dyspnea.

### Vaccination status

Information regarding the date of administration of the COVID-19 vaccine was documented through a combination of interview responses and documentation of proof of vaccine administration. Structured interviews were conducted to collect self-reported data from individuals. In cases where COVID-19 vaccination registration cards did not exist, individuals were advised to provide proof later. Individuals who chose not to use any immunization were also documented. The focus of data collection was exclusively on vaccination against COVID-19 and no assessments were carried out for other vaccinations^18^.

### Assessment of symptomatology

The assessment of the symptoms of the included individuals was based on the self-report of symptoms at the time of data collection: cough, shortness of breath or difficulty breathing, fatigue, muscle pain, headache, ageusia, anosmia, hair loss, memory loss, anxiety, runny nose^19,20^.

### Habitual physical activity—baecke questionnaire

It is a self-administered tool based on self-reporting that evaluates physical activity undertaken in the preceding 12 months. Comprising 16 items, it is categorized into three domains: occupational (items 1 to 8), sports (items 9 to 12), and leisure (items 13 to 16). Response scores follow the Likert Scale (1–5)^21^. The occupational domain score is derived by summing the indicated responses and dividing by 8 (for item 2, the indicated value should be subtracted by 6). The sports domain score is computed by summing the indicated values and dividing by 4. To calculate the leisure domain score, all selected responses are totaled, and the sum is divided by 4 (for item 13, the indicated value should be subtracted by 6). Each domain’s final score ranges from 1 to 5, with higher scores reflecting greater levels of physical activity^21,22^.

### Mini mental state examination (MMSE)

The MMSE was administered by an experienced examiner, in the form of an interview to track the individual’s cognitive function. The test aims to evaluate different cognitive parameters, containing questions that are grouped into 7 categories, and aims to evaluate specific cognitive functions such as: temporal orientation (5 points), spatial orientation (5 points), registration of three words (3 points), attention and calculation (5 points), recall of the three words (3 points), language (8 points) and visual constructive capacity (1 point). The MMSE score ranges from 0 (highest degree of cognitive impairment) to 30 points (best cognitive ability)^23^.

### Heart rate variability assessment

The morning evaluations of individuals were conducted to minimize circadian cycle variations. Sleep, diet, and room lighting were carefully controlled. All participants were instructed to maintain a regular sleep routine in the 24 h prior to data collection, with a minimum of 7 h of continuous sleep. The diet was standardized, with participants instructed to avoid meals in the 2 h prior to data collection. During the procedure, the room lighting was kept constant, preventing exposure to bright light that could alter the participants’ circadian rhythms. The individuals were instructed to refrain from consuming stimulant substances (alcohol, caffeine, nicotine, chocolate, soda, energy drinks) and practicing strenuous exercise both the day before and on the day of the exam. They were placed in a dorsal decubitus position, with temperature maintained between 22 and 24 °C and relative humidity between 50 and 60%, and instructed to stay relaxed, breathe spontaneously, refrain from speaking, and avoid any movement or sleep during the data collection process. Before data collection, they remained for a 10-min relaxation period to stabilize their heart rate^24^. The Polar RS800CX heart rate monitor (Polar Electro Oy Inc., Kempele, Finland) was employed to capture the HRV signal for a duration of 10–15 min at a sampling frequency of 1000 Hz^24^. The examiner had a minimum experience of 60 months in the analysis of the HRV signal.

Kubios standard software [HRV analysis, version 3.5.0, University of Eastern Finland, https://www.kubios.com/] was used for the analysis of HRV signals. The artifacts found were corrected using the low filter available in the software. The R–R interval series underwent detrending using the smoothing method with a set lambda of 500 and cubic interpolation at the default rate of 4 Hz. Next, a visual inspection was carried out at the collection recording time and a selection of the segment with the greatest signal stationarity in a 5-min time window. To this end, the selection met the following criteria: (1) absence of large R-R outlier intervals; (2) equidistance of R–R intervals; (3) Gaussian distribution observed in R–R intervals and heart rate graphs^25^.

The HRV analysis variables were extracted from linear (time and frequency domain) and non-linear analysis. In the time domain linear analysis, the following variables were assessed: the average R–R interval duration (Mean RR) in ms the standard deviation of all normal N-N intervals (SDNN) in ms, the average heart rate in beats per minute (bpm) the square root of successive mean squared differences of RR (RMSSD) in ms, the integral of the density of the RR interval histogram divided by its height (RR Tri) and the baseline width of a histogram displaying NN intervals (TINN)^26,27^. For the linear analysis in the frequency domain, the spectral analysis was performed by the Fast Fourier Transform (FFT) and the components were reported in high frequency (HF) (0.15–0. 4 Hz) and low frequency (LF) (0.04–0.15 Hz) in normalized units (n.u) and in milliseconds squared (ms^2^)^26,27^.

Nonlinear analysis of HRV was performed to obtain the standard deviation perpendicular to the line of identity (SD1), plot the standard deviation along the line of identity (SD2) (representing parasympathetic and sympathetic modulation, respectively), approximate entropy (ApEn), sample entropy (SampEn) and HRV fluctuation analysis detrended that describes short (DFα1) and long-term fluctuations (DFα2) where the value one (1) indicates chaotic behavior, one and a half (1.5) corresponds to regularity and a half (0.5) corresponds to randomness^27,28^.

### Statistical analysis

Quantitative data were presented as mean and standard deviation while qualitative data were presented as absolute values and percentages. The Shapiro-Wilk test was used to verify data normality. For comparisons between groups, ANOVA one way test was used when normality assumptions were met. The Kruskal–Wallis test was used when this assumption was violated. The Chi-square (χ^2^) test was used to compare qualitative data. Due to the multiple comparisons between the groups, the Tukey or Dunn’s test correction was used. Comparisons of HRV indices between groups were expressed as mean, standard deviation (SD), mean difference (MD), confidence interval of difference (95%CI).

The effect size was calculated based on the Cohen d (parametric distribution) or eta square (η^2^) (non-parametric distribution), according to the website: <https://www.psychometrica.de/effect_size.html>. The following interpretation was considered for Cohen’s d: 0.2 (small), 0.5 (moderate), and > 0.8 (large) effect size^29^. For η^2^, values close to 0.01 are considered small, those around 0.06 are moderate, and values greater than 0.15 are large effect sizes^30^. To define predictive variables for RMSSD (ms), SDNN (ms), HF (n.u.) and SD1 (ms), univariate linear regression analysis was run. Variables with a *p* < 0.20 in univariate analysis were allocated to multiple linear regression using the forward selection method^31^. The final multiple regression model was then adjusted by enter method to improve the explanation of variance (adjusted R^2^).

The homoscedasticity of the residuals was assessed by means of a scatter plot of the unstandardized residuals in relation to the fitted values. This assessment was performed to verify the assumption that the variance of the residuals is constant across the fitted values and to ensure the homoscedasticity assumption required for regression analysis^32^. We used the Durbin–Watson test to check for the presence of autocorrelation in the residuals of our regression model, where values close to 2 indicate no autocorrelation, values < 2 suggest positive autocorrelation, and values > 2 suggest negative autocorrelation^33^. Collinearity between independent variables was assessed using the Variance Inflation Factor (VIF) and tolerance: VIF below 10 and tolerance close to 1 were considered acceptable to rule out the presence of collinearity^34^. We considered a number of 15 participants for each independent variable added to the final multiple regression model^32,35^. We use the Statistical Package for the Social Sciences to performance all analysis (SPSS) [IBM—SPSS, version 20.0 for Windows, Armonk, NY, https://www.ibm.com/br-pt/]. The probability of type 1 error occurrence was established at 5% for all tests (*p* < 0.05).

## Results

**Fig. 1:**
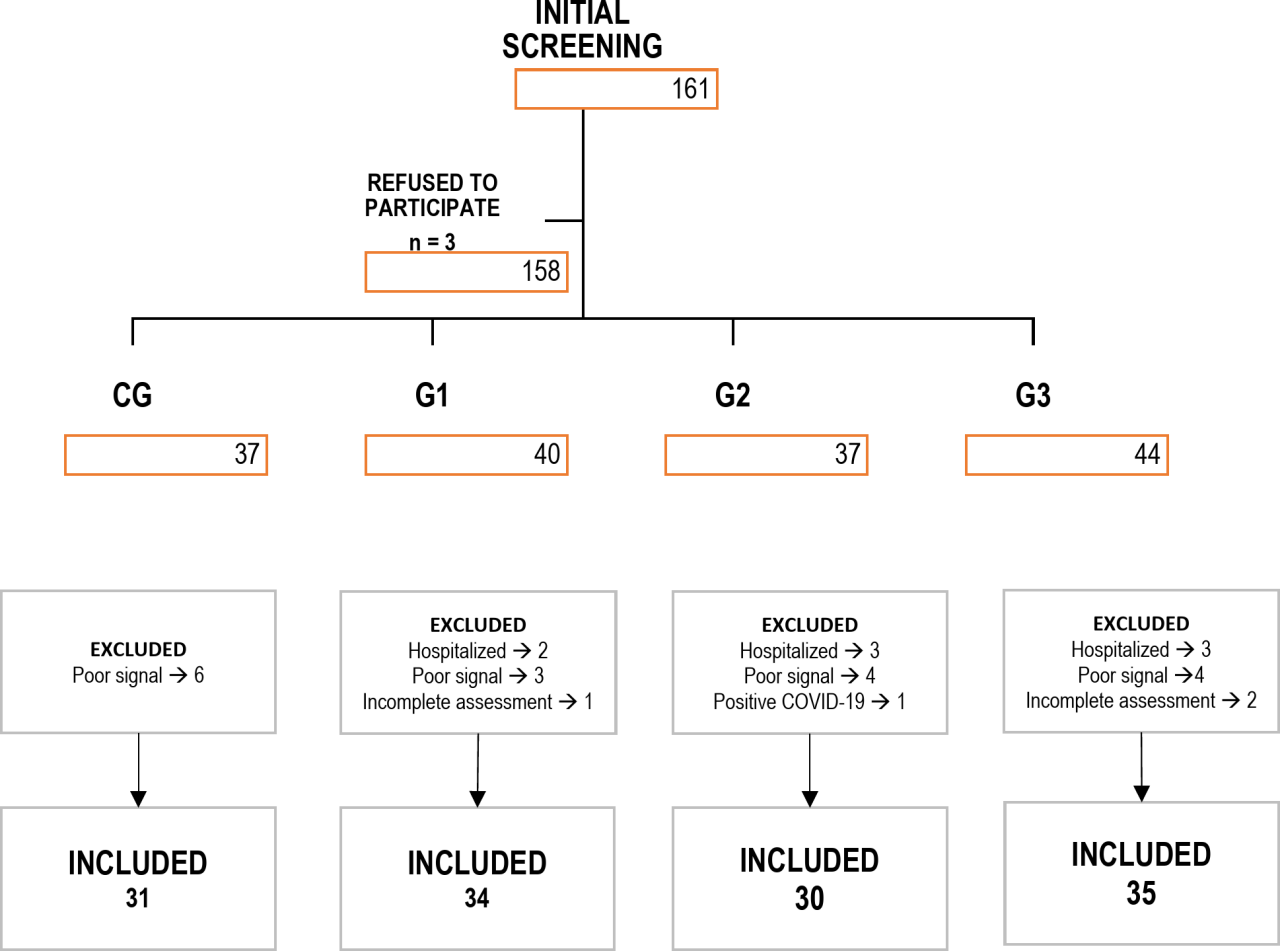
Flowchart study design including inclusion/exclusion criteria. * CG* control group, * G1* ≤6 weeks of infection, * G2* group of 2 to 6 months of infection, * G3* Group of 7 to 12 months of infection.

Initially, 161 individuals were recruited for the study. The Fig. 1 shows the flowchart of the selection criteria. According to the eligibility criteria, 31 individuals were excluded. The general and clinical characteristics of the individuals included in the study are described in Table 1. A total of 130 individuals were included: CG *n* = 31, G1 *n* = 34, G2 *n* = 30, and G3 *n* = 35. The total sample was predominantly female in groups CG (55%), G1 (62%) and G3 (77%), with the exception of G2 in which the sample had a greater number of male participants (57%). The groups are similar according to age (years), body mass (kg), height (m), BMI (kg/m^2^) and absence of cognitive deficit. The results of Baecke’s questionnaire demonstrate that the groups had a similar level of physical activity. G1 is mostly hypertensive (35%) and obese (41%) when compared to the other groups, while G2 is mostly dyslipidemic (27%).

**Table 1:** Personal and clinical characteristics of individuals included in the study (*n* = 130).

Variables	CG (*n* = 31)	G1 (*n* = 34)	G2 (*n* = 30)	G3 (*n* = 35)	*p* value
Age (years)	31 ± 10	35 ± 12	37 ± 12	33 ± 13	0.142
Gender					
Male	14 (45)	13 (38)	17 (57)	8 (23)	0.043*
Female	17 (55)	17 (62)	21 (43)	13 (77)	
Body mass (kg)	68.48 ± 13.99	78.60 ± 17.20	80.01 ± 18.99	70.98 ± 21.15	0.081
Height (m)	1.65 ± 0.09	1.69 ± 0.10	1.68 ± 0.10	1.63 ± 0.11	0.057
BMI (kg/m^2^)	24.79 ± 3.36	27.46 ± 5.51	27.75 ± 5.30	26.79 ± 7.37	0.164
Normal	16 (52)	15 (44)	9 (30)	14 (40)	0.064
Overweight	12 (38)	5 (15)	12 (40)	12 (34)	
Obesity class I	3 (10)	10 (29)	6 (20)	5 (14)	
Obesity class II	0 (0)	4 (12)	2 (7)	1 (3)	
Obesity class III	0 (0)	0 (0)	1 (3)	3 (9)	
Mini Mental State Examination	27 ± 2	26 ± 3	27 ± 3	27 ± 3	0.826
Level of physical activity					
Occupational domain	2.61 ± 0.40	2.88 ± 0.53	2.70 ± 0.62	2.73 ± 0.59	0.266
Sports domain	2.11 ± 0.55	2.57 ± 0.76	2.38 ± 0.69	2.11 ± 0.68	0.054
Leisure domain	2.71 ± 0.61	2.34 ± 0.71	2.58 ± 0.61	2.54 ± 0.71	0.146
Final score	7.43 ± 0.98	7.79 ± 1.29	7.66 ± 1.12	7.38 ± 1.30	0.327
Comorbidities					
Systemic arterial hypertension	0	12 (35)	7 (23)	3 (9)	0.001*
Stress	16 (52)	14 (42)	12 (40)	14 (40)	0.748
Thyroid dysfunction	0 (0)	1 (3)	2 (7)	1 (3)	0.515
Dyslipidemia	0 (0)	5 (15)	8 (27)	5 (14)	0.027*
Obesity	3 (10)	14 (41)	9 (30)	9 (26)	0.039*
Diabetes mellitus	0 (0)	0 (0)	3 (10)	2 (6)	0.385
Medications					
ACE inhibitor	0 (0)	9 (26)	7 (23)	2 (6)	0.041*
Levothyroxine	0 (0)	1 (3)	2 (7)	1 (3)	0.027*
Statins	0 (0)	3 (9)	7 (23)	3 (9)	0.044*
Oral hypoglycemic agents	0 (0)	0 (0)	3 (10)	2 (6)	0.385
mMRC					
0	–	1 (3)	17 (57)	24 (68)	
1	–	12 (35)	7 (23)	9 (26)	
2	–	6 (18)	3 (10)	1 (3)	< 0.001*
3	–	13 (38)	2 (7)	0 (0)	
4	–	2 (6)	1 (3)	1 (3)	
Symptomatology					
Cough	–	16 (47)	1 (3)	0 (0)	< 0.001*
Shortness of breath	–	13 (38)	1 (3)	2 (6)	< 0.001*
Fatigue	–	17 (50)	4 (13)	7 (20)	0.002*
Muscle pain	–	10 (30)	3 (10)	6 (17)	0.134
Headache	–	19 (56)	3 (10)	0 (0)	< 0.001*
Ageusia	–	18 (53)	3 (10)	1 (3)	< 0.001*
Anosmia	–	12 (35)	2 (6)	0 (0)	< 0.001*
Hair loss	–	8 (24)	1 (3)	0 (0)	0.001*
Memory loss	–	8 (24)	1 (3)	1 (3)	0.006*
Anxiety		21 (62)	4 (13)	3 (9)	< 0.001*
Runny nose	–	17 (50)	0 (0)	1 (3)	< 0.001*
Vaccination Status					
No	–	15 (44)	0 (0)	0 (0)	< 0.001*
Yes					
One dose	–	0 (0)	7 (23)	5 (14)	
Two doses	–	1 (3)	14 (46)	23 (66)	0.213
Three doses	–	18 (56)	9 (30)	7 (20)	

Data presented as mean ± standard deviation or absolute value and perceptual;* kg* kilo, * m* meter, * mMRC* modified medical research council, * ACE* angiotensin-converting enzyme, * CG* control group, * G1* ≤6 weeks of infection, * G2* group of 2 to 6 months of infection, * G3* Group of 7 to 12 months of infection. *p < 0.05 for Anova one way, Kruskal–Wallis or Chi-square test.

The groups differed according to the assessment of dyspnea (mMRC scale) and, not surprisingly, G1 had a higher percentage of individuals with functional limitations due to the presence of this symptom. Furthermore, G1 has a higher percentage of individuals with self-reported symptoms, such as cough (47%), fatigue (50%), headache (56%), ageusia (53%), anxiety (62%), runny nose (50%), and higher prevalence of individuals who had not been vaccinated (44%).

**Fig. 2:**
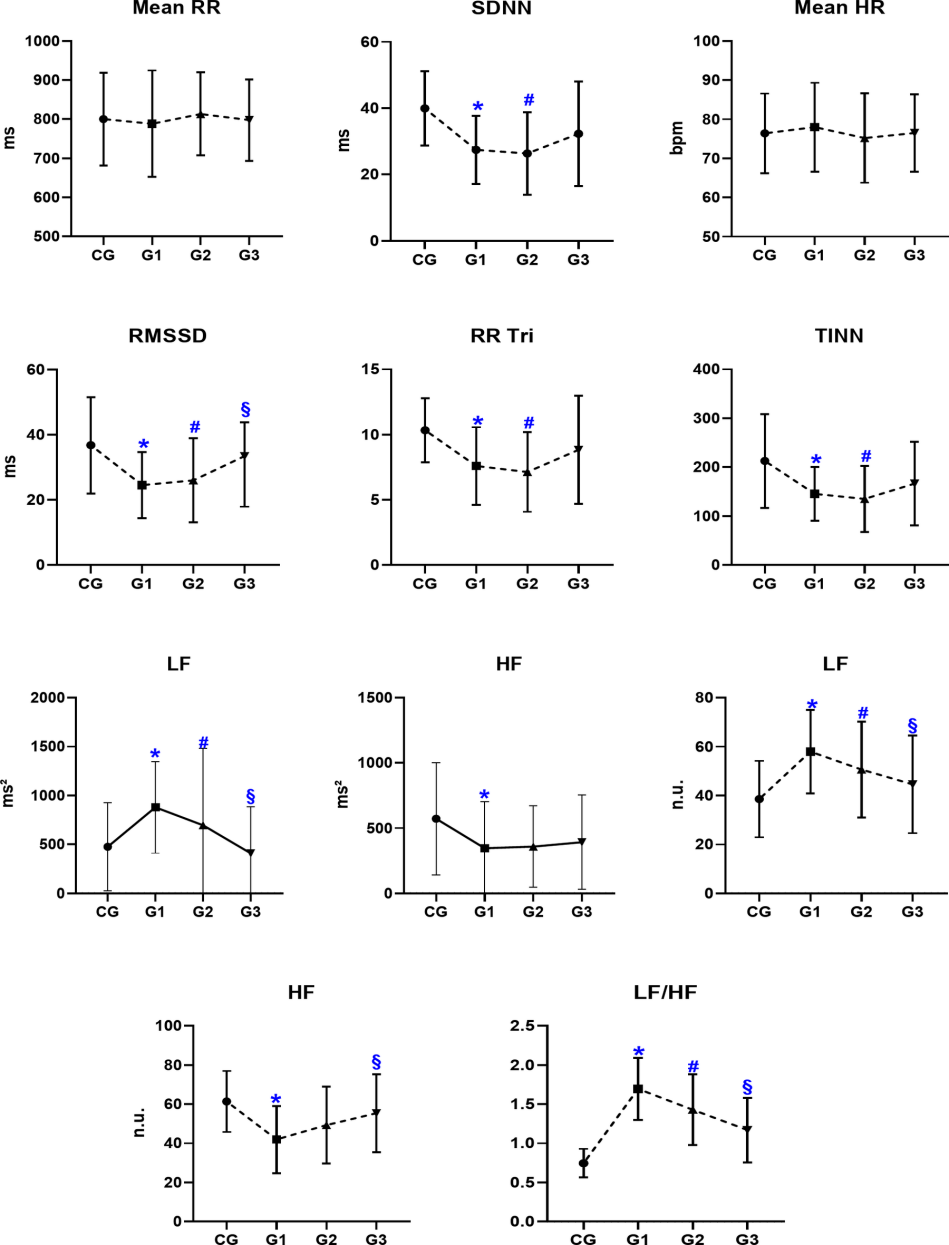
Comparative analysis of linear methods measures (time and frequency domain). Data presented as mean ± standard deviation.* Ms* milliseconds, * bpm* beats per minute, * n.u.* normalized units, * Mean RR* the average R-R interval duration, * SDNN* standard deviation of all normal N-N intervals, * Mean HR* the average heart rate, * RMSSD* the square root of successive mean squared differences of RR, * RR Tri* the integral of the density of the RR interval histogram divided by its height, * TINN* baseline width of a histogram displaying NN intervals, * LF* low frequency, * HF* high frequency, * LF/HF* ratio of low frequency by high frequency, * CG* control group, * G1* ≤6 weeks of infection, * G2* group of 2 to 6 months of infection, * G3* Group of 7 to 12 months of infection. *p < 0.05 CG versus G1; ^#^*p* < 0.05 CG versus G2; ^§^*p* < 0.05 G1 versus G3 for Anova one way or Kruskal–Wallis test.

A detailed description of the HRV indices is presented in Table 2. The results of the comparative analyzes on linear HRV measurements are shown in Fig. 2 (time and frequency domain). The SDNN, RR Tri and TINN were significantly lower in G1 and G2 when compared to the CG, suggesting a lower HRV, and indicating a possible autonomic imbalance, with possible sympathetic predominance and/or reduced parasympathetic activity (*p* < 0.05). The RMSSD (ms) and HF (n.u), strongly influenced by parasympathetic modulation, presented lower values in groups G1 and G2 when compared to the CG and, additionally, G3 presented a greater values when compared to G1 (*p* < 0.05) while only G1 showed a significant reduction in HF (ms^2^) when compared to CG. The LF n.u. and ms^2^ and the LF/HF ratio were significantly higher in groups G1 and G2 when compared to the CG group, however G3 showed a decrease in these parameters in relation to G1 (*p* < 0.05). Although LF is influenced by both sympathetic and parasympathetic activity, the observed elevation suggests a greater sympathetic contribution to heart rate control. Furthermore, the higher LF/HF ratio reinforces the predominance of sympathetic over parasympathetic activity in these groups.

**Table 2:** Descriptive analysis of resting HRV indices of individuals included in the study.

HRV indices	CG (*n* = 31)	G1 (*n* = 34)	G2 (*n* = 30)	G3 (*n* = 35)
Time domain				
Mean RR (ms)	800.30 ± 118.70	788.30 ± 136.00	813.20 ± 106.10	797.60 ± 104.00
SDNN (ms)	39.93 ± 11.14	27.41 ± 10.23	26.32 ± 12.40	32.27 ± 15.80
Mean HR (bpm)	76.41 ± 10.17	77.98 ± 11.35	75.20 ± 11.43	76.47 ± 9.92
RMSSD (ms)	36.76 ± 14.76	24.51 ± 10.10	25.99 ± 12.93	35.44 ± 20.75
RR Tri	10.33 ± 2.45	7.59 ± 2.98	7.13 ± 3.05	8.83 ± 4.16
TINN	212.80 ± 96.16	145.60 ± 54.77	135.20 ± 67.39	166.70 ± 85.08
Frequency domain				
LF (ms^2^)	476.30 ± 450.40	878.80 ± 468.20	696.50 ± 785.20	409.70 ± 476.70
HF (ms)^2^	572.10 ± 429.90	346.20 ± 355.10	357.90 ± 312.80	392.00 ± 361.20
LF (n.u.)	38.57 ± 15.57	58.00 ± 17.11	50.66 ± 19.59	44.57 ± 19.92
HF (n.u.)	61.35 ± 15.58	41.93 ± 17.12	49.27 ± 19.62	55.34 ± 19.94
LF/HF	0.75 ± 0.49	1.69 ± 1.14	1.43 ± 1.21	1.17 ± 1.20
Nonlinear analysis				
SD1 (ms)	49.82 ± 13.04	17.36 ± 7.15	18.41 ± 9.16	25.10 ± 14.70
SD2 (ms)	26.03 ± 10.45	37.73 ± 17.76	32.09 ± 15.59	34.35 ± 13.42
Apen	1.18 ± 0.06	1.13 ± 0.11	1.07 ± 0.07	1.08 ± 0.07
SampEn	1.74 ± 0.22	1.62 ± 0.31	1.75 ± 0.28	1.77 ± 0.22
DFA α1	1.15 ± 0.22	0.92 ± 0.25	1.01 ± 0.27	1.10 ± 0.27
DFA α2	0.40 ± 0.13	0.38 ± 0.16	0.36 ± 0.15	0.36 ± 0.15

*HRV* heart rate variability, * CG* control group, * G1* ≤6 weeks of infection, * G2* group of 2 to 6 months of infection, * G3* Group of 7 to 12 months of infection, * Mean RR* average R-R interval duration between heartbeats, * ms* milliseconds, * SDNN* standard deviation of RR intervals, * Mean HR* average heart rate, * bpm* in beats per minute, * RMSSD* root mean square differences of successive RR intervals, * RR Tri* integral of the RR intervals histogram divided by the height of the histogram, * TINN* triangular interpolation of NN interval histogram, * LF* normalized unit in the low-frequency band, * HF* normalized unit in the high-frequency band, * SD1* standard-deviation of the instant beat-to-beat variability, * SD2* long-term standard-deviation of continuous RR intervals, * ApEn* approximate entropy, * SampEn* sample entropy, * DFA α1* purified trend fluctuations (short-term scale), * DFA α2* purified trend fluctuations (long-term scale).

**Fig. 3:**
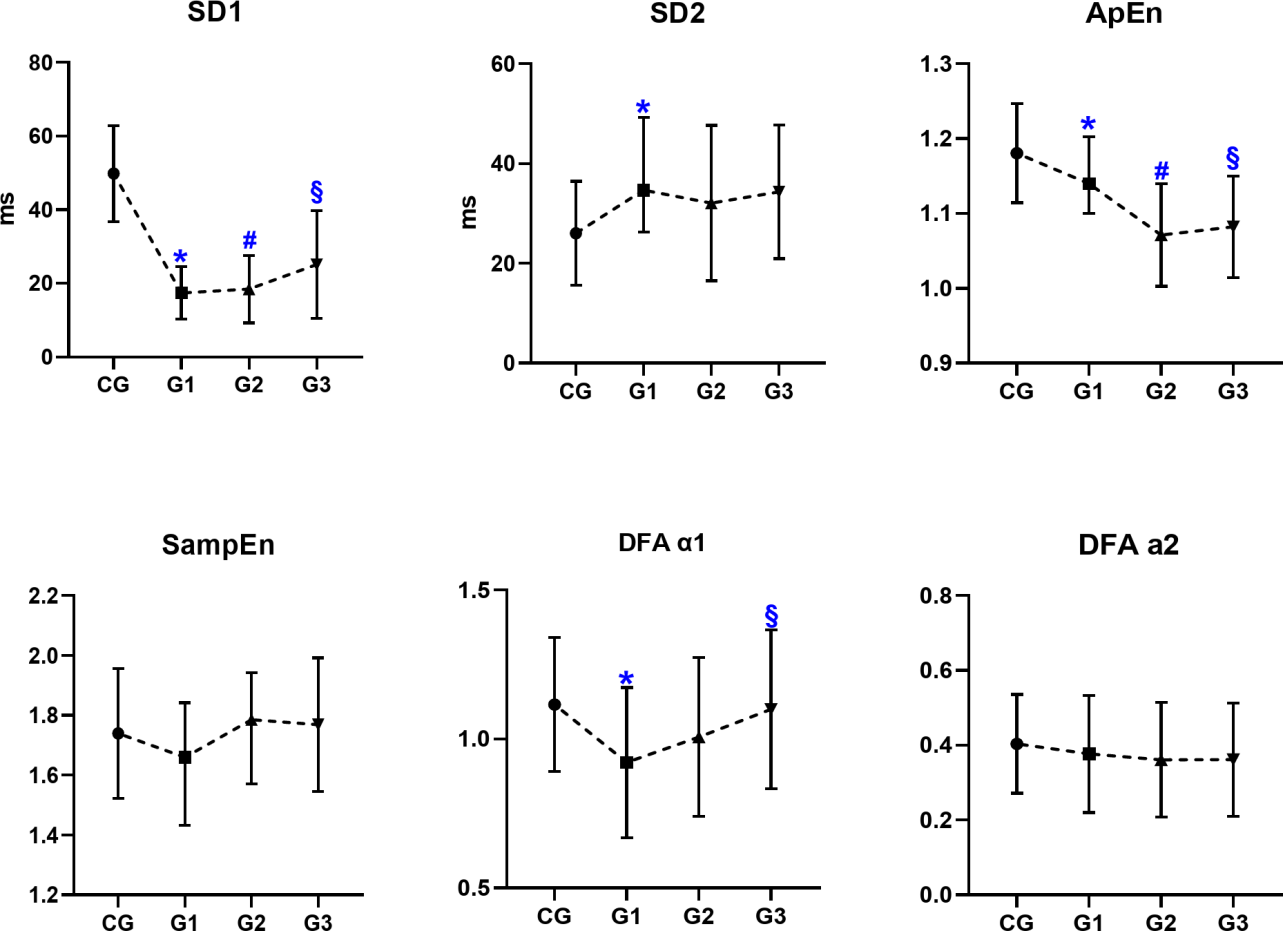
Comparative analysis of non-linear methods measures. Data presented as mean ± standard deviation. *Ms* milliseconds, * SD1* the standard deviation perpendicular to the line of identity, * SD2* the standard deviation along the line of identity, * ApEn* approximate entropy, * SampEn* sample entropy, * DFα1* analysis detrended that describes short-term fluctuations, * DFα2* analysis detrended that describes long-term fluctuations, * CG* control group, * G1* ≤6 weeks of infection, * G2* group of 2 to 6 months of infection, * G3* Group of 7 to 12 months of infection. *p < 0.05 CG versus G1; ^#^*p* < 0.05 CG versus G2; ^§^*p* < 0.05 G1 versus G3 for Anova one way or Kruskal–Wallis test.

Concerning non-linear analysis (Fig. 3), the variables SD1 and ApEn were significantly lower in groups G1 and G2 when compared to CG (*p* < 0.05), however, G3 showed an increase in this modulation when compared to G1 (*p* < 0.05). The SD1 It reflects short-term HRV and is more associated with parasympathetic activity, while ApEn quantifies the degree of regularity or determinism in the HRV signal. High ApEn values ​​indicate greater irregularity and complexity, reflecting a greater ability to adapt to varying stimuli. SD2 was higher in G1 when compared to CG (*p* < 0.05). In Table 3 we demonstrate the effect size based on Cohen’s d or η^2^ according to the magnitude of differences between groups. Moderate to large effect sizes were observed for the variables with statistically significant differences in the comparisons.

**Table 3:** Comparison of the HRV indices between groups: mean difference, 95% CI and Cohen d.

Variable	Group	Mean difference	95% CI	Effect size
Time domain				
Mean RR (ms)^#^	CG × G1	12.00	−63.63, 87.75	0.005
	CG × G2	−12.90	−90.97, 65.15	0.023
	G1 × G3	−9.20	−82.68, 64.11	0.014
SDNN (ms)^#^	**CG × G1**	**12.52**	**4.37**, ** 20.67**	**0.275**
	**CG × G2**	**13.61**	**3.87**, ** 20.67**	**0.247**
	G1 × G3	7.66	−12.77, 8.03	0.010
Mean HR (bpm)^#^	CG × G1	−1.57	−8.50, 5.37	0.145
	CG × G2	1.21	−5.93, 8.36	0.112
	G1 × G3	1.51	−5.22, 8.22	0.142
RMSSD (ms)^#^	**CG × G1**	**12.25**	**2.66**, ** 21.82**	**0.211**
	**CG × G2**	**10.77**	**0.89**, ** 20.65**	**0.130**
	**G1 × G3**	**−10.93**	**−19.00**, ** −0.29**	**0.051**
RR Tri^#^	**CG × G1**	**2.74**	**0.64**, ** 4.84**	**0.198**
	**CG × G2**	**3.20**	**1.04**, ** 5.37**	**0.251**
	G1 × G3	−1.24	−3.28, 0.79	0.012
TINN^#^	**CG × G1**	**67.20**	**17.23**, ** 117.30**	**0.227**
	**CG × G2**	**77.60**	**26.05**, ** 129.20**	**0.228**
	G1 × G3	−21.10	−69.58, 27.44	0.008
Frequency domain				
LF (ms)^#^	**CG × G1**	**−402.50**	**−781.30**, ** −23.57**	**0.225**
	**CG × G2**	**−220.20**	**−587.60**, ** 147.20**	**0.002**
	**G1 × G3**	**469.10**	**78.27**, ** 859.80**	**0.273**
HF (ms)^#^	**CG × G1**	**225.90**	**−11.50**, ** 463.20**	**0.107**
	CG × G2	214.20	−30.62, 459.00	0.090
	G1 × G3	−45.80	−276.00, 184.3	0.004
LF (n.u.)^§^	**CG × G1**	**−19.43**	**−31.17**, ** −7.69**	**1.185**
	**CG × G2**	**−12.09**	**−24.20**, ** 0.01**	**0.685**
	**G1 × G3**	**13.43**	**2.04**, ** 24.81**	**0.722**
HF (n.u.)^§^	**CG × G1**	**19.42**	**7.67**, ** 31.18**	**1.184**
	CG × G2	12.08	−0.04, 24.20	0.683
	**G1 × G3**	**−13.41**	**−24.80**, ** −2**, **01**	**0.721**
LF/HF^#^	**CG × G1**	**−0.94**	**−2.18**, ** −0.42**	**0.262**
	**CG × G2**	**−0.68**	**−1.59**, ** 0.23**	**0.125**
	**G1 × G3**	**0.52**	**0.02**, ** 1.73**	**0.105**
Non-linear analyse				
SD1^§^	**CG × G1**	**32.46**	**25.05**, ** 39.87**	**0.723**
	**CG × G2**	**31.41**	**23.76**, ** 39.06**	**0.700**
	**G1 × G3**	**−7.74**	**−14.93**, ** −0.55**	**0.051**
SD2^#^	**CG × G1**	**−11.70**	**−21.10**, ** −2.30**	**0.105**
	CG × G2	−6.06	−15.82, 3.69	0.021
	G1 × G3	3.38	−5.79, 12.56	0.003
ApEn^#^	**CG × G1**	**0.05**	**−0.151**, ** 0.046**	**0.059**
	**CG × G2**	**0.11**	**−0.102**, ** 1.14**	**0.269**
	**G1 × G3**	**0.05**	**0.055**, ** 0.164**	**0.099**
SampEn^§^	CG × G1	0.12	−0.444, 0.291	0.032
	CG × G2	−0.01	−0.185, 0.161	0.002
	G1 × G3	−0.15	−0.314, 0.011	0.064
DFA α1^§^	**CG × G1**	**0.23**	**0.032**, ** 0.357**	**0.804**
	CG × G2	0.14	−0.060, 0.277	0.407
	**G1 × G3**	**−0.18**	**−0.337**, ** −0.020**	**0.706**
DFA α2^#^	CG × G1	0.02	−0.069, 0.123	0.008
	CG × G2	0.04	−0.057, 0.142	0.018
	G1 × G3	0.02	−0.078, 0.109	0.003

Data presented as mean difference, 95% confidence interval and effect size via Cohen’s d.* CG* control group, * G1* ≤6 weeks of infection, * G2* group of 2 to 6 months of infection, * G3* Group of 7 to 12 months of infection, * ms* milliseconds, * bpm* beats per minute, * n.u.* normalized units, * Mean RR* the average R-R interval duration, * SDNN* standard deviation of all normal N-N intervals, * Mean HR* the average heart rate, * RMSSD* the square root of successive mean squared differences of RR, * RR Tri* the integral of the density of the RR interval histogram divided by its height, * TINN* baseline width of a histogram displaying NN intervals, * LF* low frequency, * HF* high frequency, * LF/HF* ratio of low frequency by high frequency, * SD1* the standard deviation perpendicular to the line of identity, * SD2* the standard deviation along the line of identity, * ApEn* approximate entropy, * SampEn* sample entropy, * DFα1* analysis detrended that describes short-term fluctuations, * DFα2* analysis detrended that describes long-term fluctuations, * SD1* the standard deviation perpendicular to the line of identity, * SD2* the standard deviation along the line of identity, * ApEn* approximate entropy, * SampEn* sample entropy, * DFα1* analysis detrended that describes short-term fluctuations, * DFα2* analysis detrended that describes long-term fluctuations. Highlights in bold demonstrate statistical difference (*p* < 0.05). ^#^Non-parametric distribution (Kruskal–Wallis test; η^2^ effect size); ^§^Parametric distribution (ANOVA one-way test; Cohen’s d effect size).

Comprehensive assessments were analyzed to identify predictors of SDNN (ms), RMSSD (ms), HF (n.u.) and SD1 (ms) through univariate linear regression, as detailed in Table 4. Age (years), and infection time (months) were found to significantly influence SDNN (ms), RMSSD (ms) and SD1 (ms) (*p* < 0.05) while only infection time was significantly associated with HF (n.u.) (*p* < 0.05).

**Table 4:** Univariate regression analysis of factors potentially associated with overview parameters of HRV and clinical outcomes.

Variables	Non-standard coefficients		Standard coefficients	t	*P* value	Adjusted *R*^2^
	β	Error	β			
RMSSD (ms)						
Infection time (month)	1.205	0.344	0.337	3.508	0.001*	0.104
Sex (0, female; 1, male)	3.588	2.783	0.114	1.289	0.200	0.005
Age (years)	−0.545	0.106	−0.414	−5.124	< 0.001*	0.165
BMI (kg/m^2^)	−0.288	0.240	−0.106	−1.199	0.233	0.003
Systemic arterial hypertension (0, no; 1, yes)	−4.006	3.636	−0.097	−1.102	0.273	0.002
Stress (0, no; 1, yes)	−2.372	2.770	−0.076	−0.856	0.393	−0.002
Thyroid dysfunction (0, no; 1, yes)	0.980	7.926	0.011	0.124	0.902	−0.008
Dyslipidemia (0, no; 1, yes)	−1.314	3.964	−0.029	−0.331	0.741	−0.007
Obesity (0, no; 1, yes)	−3.311	3.105	−0.094	−1.066	0.288	0.001
Diabetes mellitus (0, no; 1, yes)	−0.669	6.065	−0.010	−0.110	0.912	−0.008
mMRC (0, 1, 2, 3, 4)	−1.588	1.258	−0.128	−1.262	0.210	0.006
SDNN (ms)						
Infection time (month)	0.589	0.303	0.193	1.941	0.045*	0.027
Sex (0, female; 1, male)	1.908	2.406	0.070	0.793	0.429	−0.003
Age (years)	−0.519	0.090	−0.455	−5.781	< 0.001*	0.201
BMI (kg/m^2^)	−0.302	0.207	−0.128	−1.464	0.146	0.009
Systemic arterial hypertension (0, no; 1, yes)	−5.668	3.111	−0.159	−1.822	0.071	0.018
Stress (0, no; 1, yes)	0.456	2.386	0.017	0.191	0.849	−0.008
Thyroid dysfunction (0, no; 1, yes)	0.336	6.842	0.004	0.049	0.961	−0.008
Dyslipidemia(0, no; 1, yes)	−4.427	3.399	−0.114	−1.302	0.195	0.005
Obesity (0, no; 1, yes)	−2.804	2.652	−0.093	−1.057	0.292	0.001
Diabetes mellitus (0, no; 1, yes)	−2.060	5.232	−0.035	−0.394	0.694	−0.007
mMRC (0, 1, 2, 3, 4)	−0.834	1.082	−0.078	−0.770	0.443	−0.004
HF (n.u.)						
Infection time (month)	1.462	0.437	0.322	3.345	0.001*	0.094
Sex (0, female; 1, male)	4.284	3.461	0.109	1.238	0.218	0.004
Age (years)	−0.188	0.144	−0.114	−1.304	0.195	0.005
BMI (kg/m^2^)	−0.203	0.300	−0.060	−0.676	0.500	−0.004
Systemic arterial hypertension (0, no; 1, yes)	−2.231	4.545	−0.043	−0.491	0.624	−0.006
Stress (0, no; 1, yes)	−5.803	3.406	−0.149	−1.704	0.091	0.015
Thyroid dysfunction (0, no; 1, yes)	2.517	9.874	0.023	0.255	0.799	−0.007
Dyslipidemia(0, no; 1, yes)	−5.113	4.918	−0.092	−1.040	0.300	0.001
Obesity (0, no; 1, yes)	−3.580	3.832	−0.082	−0.934	0.352	−0.001
Diabetes mellitus (0, no; 1, yes)	−1.300	7.556	−0.015	−0.172	0.864	−0.008
mMRC (0, 1, 2, 3, 4)	−1.474	1.614	−0.092	−0.913	0.364	−0.002
SD1 (ms)						
Infection time (month)	0.954	0.251	0.360	3.806	< 0.001*	0.121
Sex (0, female; 1, male)	1.098	3.091	0.031	0.355	0.723	−0.007
Age (years)	−0.519	0.121	−0.355	−4.295	< 0.001*	0.119
BMI (kg/m^2^)	−0.482	0.264	−0.160	−1.830	0.070	0.018
Systemic arterial hypertension (0, no; 1, yes)	−9.980	3.943	−0.218	−2.531	0.074	0.040
Stress (0, no; 1, yes)	1.046	3.058	0.030	0.342	0.733	−0.007
Thyroid dysfunction (0, no; 1, yes)	−5.432	8.759	−0.055	−0.620	0.536	−0.005
Dyslipidemia(0, no; 1, yes)	−7.820	4.331	−0.158	−1.805	0.073	0.017
Obesity (0, no; 1, yes)	−6.301	3.370	−0.163	−1.870	0.064	0.019
Diabetes mellitus (0, no; 1, yes)	−6.748	6.685	−0.089	−1.009	0.315	0.001
mMRC (0, 1, 2, 3, 4)	−1.150	0.936	−0.124	−1.230	0.222	0.005

*HRV* heart rate variability, * SDNN* standard deviation of all normal N-N intervals, * ms* milliseconds, * BMI* body mass index, * kg* kilos, * m* meter, * mMRC* modified medical research council, *RMSSD* the square root of successive mean squared differences of RR, * HF* high frequency, * n.u*. normalized units, * SD1* the standard deviation perpendicular to the line of identity. *Statistical significance (*p* < 0.05).

The multiple linear regression models can be seen in Table 5. A multiple linear regression model for RMSSD indicated that both age and infection duration were significant predictors (β age = −0.456, *p* < 0.001; β infection time = 1.063, *p* = 0.001), with an adjusted R^2^ of 0.227. For SDNN age had a significant negative association (β = −0.476, *p* < 0.001), while infection duration showed no significant effect (*p* = 0.122). The model explained 21.3% of the variance (adjusted R^2^ = 0.213). The Model 2 (SDNN), which included systemic arterial hypertension, BMI (kg/m^2^), dyslipidemia, infection duration and age as additional predictors, just age remained significant predictor, while hypertension, BMI, infection time, and dyslipidemia were non-significant. This model explain 19.20% of the variance (adjusted R^2^: 0.192) For HF (n.u.), infection duration was a significant predictor in both models (*p* = 0.002), whereas age was marginally non-significant (*p* = 0.068 in Model 1; *p* = 0.055 in Model 2). Stress emerged as a significant negative predictor in Model 2 (β = −7.386, *p* = 0.049), increasing model variance explained to 14.3%. In models predicting SD1, both age and infection duration were significant (*p* < 0.001 for age; *p* = 0.001 for infection) across both models, with adjusted R^2^ values of 0.242 (Model 1) and 0.226 (Model 2). BMI, hypertension, and dyslipidemia showed no significant effects on SD1 (Model 2).

**Table 5:** Multiple linear regression to determine the influence clinical outcomes in HRV.

Model	Variables	Non-standard coefficients		t	P value	Collinearity statistics		Adjusted R^2^	ANOVA p value	Durbin–Watson
		β	Error			Tolerance	VIF			
1	RMSSD									
	Constant	38.898	4.657	8.353	< 0.001*			0.227	< 0.001*	2.065
	Infection time (month)	1.063	0.321	3.311	0.001*	0.988	1.012			
	Age (years)	−0.456	0.113	−4.037	< 0.001*	0.988	1.012			
1	SDNN									
	Constant	43.636	4.019	10.857	< 0.001*			0.213	< 0.001*	1.946
	Infection time (month)	0.429	0.275	1.562	0.122	0.986	1.014			
	Age (years)	−0.476	0.097	−4.882	< 0.001*	0.986	1.014			
2	Constant	47.898	6.968	6.874	0.000			0.192	< 0.001*	1.927
	Infection time (month)	0.430	0.284	1.515	0.133	0.948	1.055			
	Age (years)	−0.482	0.100	−4.811	0.000	0.957	1.045			
	Systemic arterial hypertension (0, no; 1, yes)	0.405	2.970	0.136	0.892	0.917	1.091			
	BMI (kg/m^2^)	−0.158	0.208	−0.763	0.448	0.867	1.153			
	Dyslipidemia (0, no; 1, yes)	1.024	3.296	0.311	0.757	0.865	1.156			
1	HF (n.u)									
	Constant	51.816	6.361	8.147	< 0.001*			0.116	0.001*	2.030
	Infection time (month)	1.367	0.435	3.142	0.002*	0.986	1.014			
	Age (years)	−0.285	0.154	−1.849	0.068	0.986	1.014			
2	Constant	55.302	6.505	8.502	< 0.001*			0.143	0.001*	2.018
	Infection time (month)	1.341	0.429	3.129	0.002*	0.985	1.015			
	Age (years)	−0.296	0.152	−1.945	0.055	0.985	1.016			
	Stress (0, no; 1, yes)	−7.386	3.711	−1.990	0.049*	0.998	1.002			
1	SD1 (ms)									
	Constant	27.880	3.426	8.137	< 0.001*			0.242	< 0.001*	2.034
	Infection time (month)	0.841	0.234	3.588	0.001*	0.986	1.014			
	Age (years)	−0.337	0.083	−4.061	< 0.001*	0.986	1.014			
2	Constant	30.029	5.927	5.066	< 0.001*			0.226	< 0.001*	1.993
	Infection time (month)	0.867	0.241	3.590	0.001*	0.948	1.055			
	Age (years)	−0.352	0.085	−4.129	< 0.001*	0.957	1.045			
	Systemic arterial hypertension (0, no; 1, yes)	1.661	2.527	0.657	0.513	0.917	1.091			
	Dyslipidemia (0, no; 1, yes)	1.921	2.804	0.685	0.495	0.865	1.156			
	BMI (kg/m^2^)	−0.091	0.177	−0.517	0.606	0.867	1.153			

*HRV* heart rate variability, * ANOVA* analysis of variance, * SDNN* standard deviation of all normal N-N intervals, * ms* milliseconds, * BMI* body mass index, * kg* kilos, * m* meter, * mMRC* modified medical research council, * RMSSD* the square root of successive mean squared differences of RR, * HF* high frequency, * n.u.* normalized units; SD1: the standard deviation perpendicular to the line of identity, * VIF* variance inflation factor. *Statistical significance (*p* < 0.05).

## Discussion

The main findings of this investigation are: (1) we confirmed our hypothesis that non-hospitalized individuals who had COVID-19 present a decrease in HRV with a predominance of the sympathetic nervous system; (2) above all, those evaluated early in the first weeks after infection have less parasympathetic modulation; (3) moderate to large effect sizes were found when comparing groups; (4) the time after diagnosis influences positively while age is negatively associated with HRV parameters.

Initially, we had highlighted an important gap that motivated us to conduct this investigation: the elementary and inconclusive nature of the studies that proposed to evaluate cardiac autonomic modulation in individuals who were affected by COVID-19 using HRV as an assessment tool. When carrying out a thorough search in the literature, we observed divergences in the evidence on this topic, that is, evidences that attests to an increase in sympathetic modulation^3,10,36,37^ in post-COVID individuals and evidences that demonstrates the opposite (greater parasympathetic modulation)^38–41^.

Our results are in line with the hypothesis that this population presents a decrease in HRV and and a tendency towards greater sympathetic modulation^38–41^. In the linear analysis, time-domain variables reflecting the global HRV, such as SDNN (ms), RMSSD (ms), RR Tri, and TINN^27,42^, showed significant reductions compared to the control group, particularly in groups with shorter recovery times. This reduction was followed by an increase in these indices among individuals with longer recovery periods. This pattern may indicate not only a reduction in global autonomic modulation, possibly due to sympathetic predominance or reduced parasympathetic activity, but also suggests a transitional phase of autonomic recovery.

In the frequency domain, the LF band, which reflects both modulations but with greater sympathetic predominance, showed a significant increase in the evaluated group during the first weeks after infection, with a tendency to decrease over time. In contrast, the HF band, which reflects parasympathetic activity, exhibited a significant reduction in those evaluated early, indicating diminished parasympathetic activity. However, no increase in this variable was observed over time. This behavior can be attributed to the different recovery dynamics of the sympathetic and parasympathetic nervous systems after stressors like infection^43^. Sympathetic modulation tends to normalize more quickly following the acute stress response, whereas parasympathetic modulation requires more time to recover, especially after inflammatory states^44^. Additionally, COVID-19 induces vagal suppression, which reduces parasympathetic activity and prolongs its recovery period^44^.

Linear HRV parameters, despite their widespread use, fail to fully capture the complexity of ANS activity, given its inherently nonlinear characteristics^45^. Nonlinear indices, therefore, provide a more comprehensive perspective by accounting for the irregularity, complexity, and dynamic properties of HRV signals. These measures tend to be more sensitive in detecting subtle changes in autonomic balance, complementing linear analysis by uncovering hidden aspects of autonomic regulation^46^. The decrease in parasympathetic modulation can also be evidenced through nonlinear analysis indices such as SD1, which showed a significant reduction compared to the control group. Despite a gradual increase over time in the recovery groups, SD1 values remained considerably lower than those of the control group. This finding suggests a persistent autonomic dysfunction, reflecting an incomplete recovery of parasympathetic modulation capacity. SD1, related to short-term variability, highlights the suppression of vagal activity, even in more advanced recovery phases^44^.

Additionally, ApEn was also temporally impaired, showing consistently lower values than the control group over the months. Clinically, this reflects a reduction in the irregularity and complexity of HRV signals^42^. Its decrease indicates a diminished adaptive capacity of the autonomic nervous system in response to physiological stress, underlining the prolonged impact of autonomic imbalance during recovery. Although the group with a shorter recovery time showed higher SD2 values, interpreting this index requires caution, as it is more closely related to long-term variations. This increase may not directly indicate functional improvement. In the context of post-COVID-19 recovery, it’s important to note that SD2, when considered alone, may not fully capture the dynamics of autonomic recovery. Therefore, it should be analyzed alongside other indices for a more comprehensive evaluation^27,42^.

When we contrasted our results to current literature, we observed that, Silva et al.^3^, when investigating the impact of long COVID on HRV, 4–16 weeks after infection, concluded that this population had a decrease in parasympathetic modulation when evaluated in the position supine and during the respiratory sinus arrhythmia maneuver. Stute et al.^10^ investigated healthy young adults 3–8 weeks after testing positive for SARS-CoV-2 and observed that these individuals may have reductions in autonomic function, supported by greater resting sympathetic activity and cardiovascular responses to orthostasis compared to healthy controls. Furthermore, Shah et al.^37^, observed that cardiovascular dysautonomia is a frequent occurrence in people who have recovered from COVID-19 (30–45 days after infection), presenting a significantly lower HRV compared to individuals healthy controls. Some points must be considered within these investigations, especially the methodological aspects: Silva et al.^3^ used a portable device to collect the biological signal, while Stute et al.^10^ and Shah et al.^37^ used a 12-lead electrocardiogram. Coincidentally, everyone performed short-term analyzes of HRV variables (< 10 min).

Before discussing the studies that attest to the increase in parasympathetic activity, we seek to understand which mechanisms can justify the express sympathetic modulation in previous studies. A recent review brings with it an approach that involves the baroreflex, the renin-angiotensin-aldosterone system and the angiotensin-converting enzyme 2 (ACE2), the latter being one of the main entry points for SARS-CoV-2 into the intracellular environment^47^. Brefly, this causes the interaction between the viral complex with ACE2 to significantly reduce the amount of ACE2 receptor bound to the membrane, leading to impaired blood pressure control and, consequently, a decrease in baroreflex sensitivity. Such a decrease can result in a reduction in parasympathetic activity and an increase in sympathetic activity, associated with the autonomic control of the cardiovascular system and reflecting on HRV^47^. Additionally, regardless of COVID-19 severity, baroreflex sensitivity is significantly reduced^48^.

The neurotropism of SARS-CoV-2 and its interaction with the central nervous system, particularly the brainstem, plays a central role in the neurological and dysautonomic manifestations associated with the infection^47^. The virus affects vital structures responsible for cardiac autonomic control, disrupting the balance between the sympathetic and parasympathetic systems, which is reflected in reduced HRV, especially in severely ill patients and during acute conditions^47^. Clinically, this behavior may be linked to the inflammatory nature of the disease^2^. Elevated levels of pro-inflammatory markers such as interleukin-6 (IL-6), tumor necrosis factor-alpha (TNF-α), and C-reactive protein are characteristic features of COVID-19 and have been strongly associated with autonomic dysfunction and this can be partly explained by the fact that the cholinergic anti-inflammatory pathway, which is vagally mediated, is impaired due to the cascade of pro-inflammatory cytokines^6,43^. A previous study demonstrated that significant reductions in HRV preceded elevations in C-reactive protein levels within 72 h in COVID-19 patients^2^. Although the relationship between the inflammatory state and HRV has been extensively studied, a specific mechanism detailing how the immune system interacts with the ANS and impacts cardiac autonomic balance remains unclear. However, what is known thus far is that HRV has an inverse relationship with inflammation^49^.

In contrast to the results found here, Haischer et al.^38^ determined whether COVID-19 survivors with a series of persistent symptoms had parasympathetic dominance of HRV even 8 months after infection. The study by Asarcikli et al.^39^ revealed a parasympathetic connotation and increased HRV in patients evaluated between 12 and 26 weeks after infection. The results of the study by Karakayalı et al.^40^ when comparing symptomatic and asymptomatic post-COVID patients 12–26 weeks after infection revealed increased parasympathetic tone and HRV in those who were symptomatic compared to those who were asymptomatic. Methodologically, all the previously mentioned studies used an electrocardiogram to collect the biological signal, however, Haisher et al.^38^ analyzed short-term HRV, while Asarcikli et al.^39^ and Karakayalı et al.^40^ long-term (24 h). The authors defend the hypothesis that the increase in cardiovascular parasympathetic expression occurs through a compensatory anti-inflammatory response through the vagal-cholinergic pathway of the parasympathetic branch and this depends on the individual’s current inflammatory state and modulation by the central nervous system^38,39^. Basically, this pathway is activated in the afferent branch of the dorsal vagal complex due to damage caused by cellular invasion by SARS-CoV-2, and causes the transcriptional factor of pro-inflammatory cytokines to be interrupted, in addition to resulting in the non-replication of the coronavirus^6^.

Although the predictive capacity in individuals with mild post-COVID conditions has not yet been explored, clinical application requires practical values, such as cut-off points to distinguish between risk and no risk for unfavorable outcomes based on HRV parameters, including RMSSD (ms), SDNN (ms), frequency domain and non-linear indices. Studies indicate that a 5-min RMSSD in the lowest quartile is associated with a 56% higher risk of mortality^50^. Additionally, low HRV is linked to a 32–45% increased risk of a first cardiovascular event in populations without known cardiovascular disease, while a 1% increase in SDNN is associated with an approximately 1% lower risk of fatal or non-fatal CVD^51^. However, defining specific thresholds for these parameters remains a scientific challenge, even in healthy populations. The largest systematic reviews with meta-analyses published to date have been unable to establish cut-off points due to the high heterogeneity of studies, limiting the applicability of these findings in defining clinical outcomes^50,51^.

Few studies have explored the predictive capacity of HRV in individuals affected by COVID-19, especially those hospitalized and with greater disease severity. In critically ill patients with a mean age of 68 ± 18 years, a long-term SDNN (24 h) cutoff point of 70 ms, with a sensitivity and specificity of 0.48 and 0.86, demonstrated predictive ability for hospital mortality within the first 24 h after admission to the intensive care unit, with an adjusted hazard ratio of 3.70 (95% CI 1.34–10.24)^52^. However, despite being a pioneering study in exploring HRV for predicting unfavorable outcomes, the study’s small sample size represents a limitation, and the findings lack generalizability: the study included only critically ill patients without associated heart diseases and without the use of inotropes or vasopressors^52^. Using ultra-short-term HRV (< 15s), an SDNN (ms) ≤ 8 was able to predict survival three weeks after hospital admission in patients aged 70 years or older (HR = 0.32, 95% CI: 0.16–0.64, *p* = 0.001). Additionally, those with an RMSSD (ms) > 8 exhibited a lower risk of being subsequently transferred from the hospital ward to the ICU (HR = 0.51, 95% CI: 0.29–0.90, *p* = 0.021)^53^.

However, this is the first study that considers individuals affected by the disease for a period of up to twelve months and, despite the basic nature of the investigation, the data obtained in this research will play an essential role in understanding cardiac autonomic changes in these individuals. Furthermore, the present study contribute to provide the opportunity for new investigations to be designed in order to answer future questions: (1) does COVID-19 temporarily impact the autonomic nervous system?; (2) does this population have greater sympathetic or parasympathetic expression? Despite the observance of HRV changes, it is not defined whether these changes are the result of direct infection by SARS-CoV-2 infection to the ANS or due to systemic inflammation. Additionally, although the sample size of the study was pre-determined and adequate to detect moderate to large effects using a power calculation, it may not have enough power to detect smaller and potentially significant differences in HRV. This could especially impact the interpretation of non-significant results. We recommend that future research consider larger sample sizes to uncover more subtle differences, particularly in nonlinear indices such as SampEn, which may reflect complex autonomic patterns.

### Clinical impact

Individuals with post-COVID have an imbalance in autonomic modulation with significant impairments in HRV. Therefore, the autonomic changes developed by COVID-19 must be considered from the beginning and accompanied with specific therapies that minimize the deleterious effects of the pathology on the autonomic nervous system. These findings may contribute to avoiding unfavorable outcomes, especially those of a cardiovascular nature, in the short and long term. Professionals involved in the rehabilitation process of this population must be alert and include measures that reflect cardiac autonomic status in their routine assessment, as this outcome can predict clinical changes. Moreover, the importance of the lowest percentiles in HRV is crucial, given that no definitive cutoff point has been established. This emphasizes the need for a comprehensive approach to monitoring autonomic function in post-COVID patients, especially in the absence of universally accepted reference thresholds.

### Limitations

Firstly, individuals were not evaluated longitudinally. This inherently limits the ability to determine causality. A longitudinal approach would offer more robust insights into the temporal recovery of autonomic function post-COVID, revealing individualized trajectories of recovery or persistence of autonomic imbalance. Future studies should be guided by specific hypotheses addressing the reversibility of observed autonomic alterations. These could include exploring how the severity of COVID-19 and vaccination status influence autonomic recovery, investigating the modulatory role of inflammatory cytokines in restoring vagal function, and evaluating the potential benefits of exercise-based cardiovascular rehabilitation programs in enhancing autonomic resilience. Secondly, we used a portable heart rate monitor to collect the biological signal, however, this method is widely validated^54,55^. Third, in the present study we consider to recruit a comprehensive form of the population affected by COVID-19 was considered (symptomatic and asymptomatic). In this context, young adults make up the majority of the included sample, and these results may not extend to older populations or those with pre-existing conditions that amplify autonomic.

## Conclusion

Post-COVID individuals show a decrease in HRV and a tendency towards greater sympathetic expression when compared to a healthy matched controls, especially those assessed in the acute phase of infection (< 6 weeks). Time since infection and age are predictors of HRV recovery, explaining up to 24.2% of this outcome and suggesting a transient effect of the disease on the autonomic nervous system. Clinically, monitoring ANS by HRV in post-COVID patients, especially in the early phases of recovery, may be useful to identify those with worse cardiac autonomic modulation, risk of cardiovascular outcomes and to direct individualized cardiovascular and pulmonary rehabilitation programs. We encourage robust longitudinal clinical trials to confirm our findings.

## Data Availability

The datasets used and/or analyzed during the current study are available from the corresponding author on reasonable request.
